# U structured network with three encoding paths for breast tumor segmentation

**DOI:** 10.1038/s41598-023-48883-y

**Published:** 2023-12-07

**Authors:** Huajie Zhang, Qianting Ma, Yunjie Chen

**Affiliations:** School of Mathematics and Statistics, Nanjing University of Information Science and Technology, Nanjing, 210044 China

**Keywords:** Breast cancer, Computer science

## Abstract

Breast ultrasound segmentation remains challenging because of the blurred boundaries, irregular shapes, and the presence of shadowing and speckle noise. The majority of approaches stack convolutional layers to extract advanced semantic information, which makes it difficult to handle multiscale issues. To address those issues, we propose a three-path U-structure network (TPUNet) that consists of a three-path encoder and an attention-based feature fusion block (AFF Block). Specifically, instead of simply stacking convolutional layers, we design a three-path encoder to capture multiscale features through three independent encoding paths. Additionally, we design an attention-based feature fusion block to weight and fuse feature maps in spatial and channel dimensions. The AFF Block encourages different paths to compete with each other in order to synthesize more salient feature maps. We also investigate a hybrid loss function for reducing false negative regions and refining the boundary segmentation, as well as the deep supervision to guide different paths to capture the effective features under the corresponding receptive field sizes. According to experimental findings, our proposed TPUNet achieves more excellent results in terms of quantitative analysis and visual quality than other rival approaches.

## Introduction

Breast cancer is a disease that poses a huge danger to women’s lives and health^1,2^. In most cases, breast cancer may typically be prevented by early screening and timely diagnosis^3–5^. Breast ultrasound (BUS) has become a popular breast screening technology due to its low cost and low risk^6,7^. Breast tumors are manually delineated by radiologists, which is challenging since it consumes time and requires specialized knowledge. Therefore, automatic breast segmentation methods based on machine learning are of great significance. Besides, the diagnosis of BUS images is extremely dependent on physician experience, which is prone to inter- and intra-rater differences^8,9^. Computer-aided diagnostic (CAD) technologies are widely used to increase the system’s dependability and help radiologists identify and diagnose breast tumors.

Machine learning methods are a burgeoning field that has been extensively employed for classification and segmentation^10^. By stacking convolutional layers, Convolutional Neural Networks (CNNs) can extract complex semantic features, producing outstanding outcomes in medical image segmentation. Long et al.^11^ replaced the fully connected layers in the traditional CNNs with $1 \times 1$ convolution, which significantly reduces computational expense. Kaiming et al.^12^ proposed a residual learning framework that improves gradient flow and information flow effectively through residual mapping. Huang et al.^13^ proposed a network with dense connections that encourages feature reuse to stimulate the potential of the network. UNet introduced a symmetrical encoder–decoder framework, which enhances the delivery of spatial information effectively^14^. Guan et al.^15^ introduced dense convolution operations to UNet and achieved excellent results in their work on removing artifacts from 2D photoacoustic tomography images. Similarly, Li et al.^16^ designed a dense convolutional network that combines 2D and 3D segmentation, which achieved high-quality 3D liver and tumor segmentation with fewer parameters.

**Figure 1:**
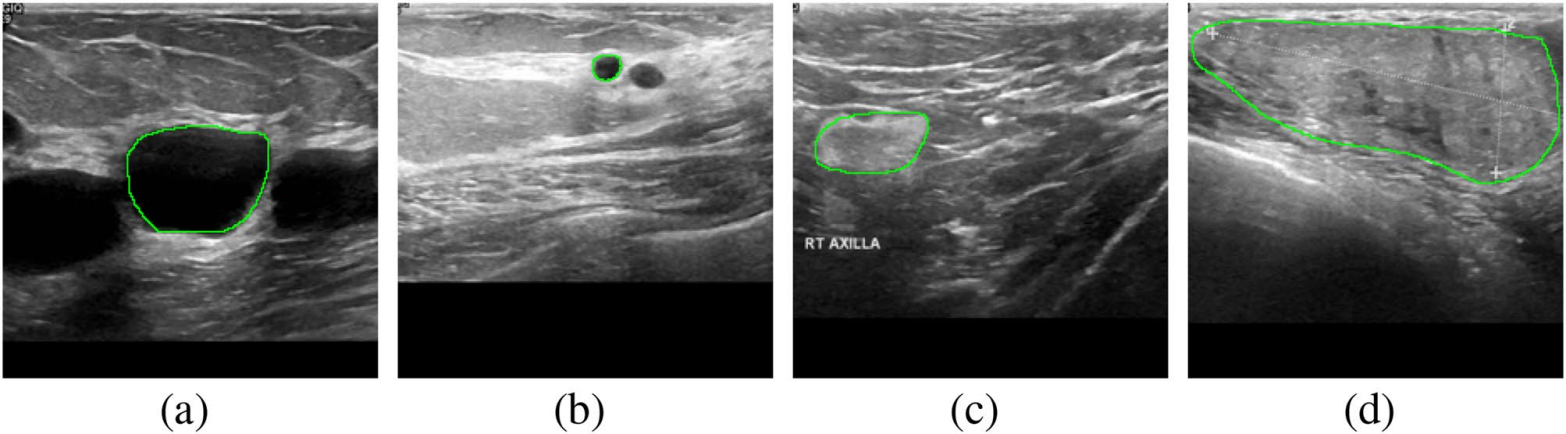
Examples show the heterogeneity in breast tumors. The green line outlines the boundary of the tumor. (**a**,**b**) Show the interference of normal benign tissue with the tumor. (**c**,**d**) Show the blurring of the boundary due to low contrast, which makes it difficult to segment.

The classical encoder–decoder network offers various benefits for medical image segmentation. For example, it contains plenty of convolutional and pooling operations, which filter noise effectively by repeatedly extracting features. The symmetrical structure enhances the transmission of spatial information, which increases the accuracy of prediction. In addition, multichannel feature extraction shows excellent performance in removing artifacts. However, the architecture of a single encoder makes it hard to extract multiscale features. In breast ultrasound segmentation, as shown in Fig. 1, the size, shape, location, and grayscale intensity of breast tumors vary greatly due to individual variances and breast tissue deformation, which requires a high level of multiscale feature extraction capability. In addition, some benign lesions and normal tissue structures resemble breast tumors, which may lead to segmentation errors. Low contrast between breast cancer and normal tissue causes indistinct borders and makes it difficult to segment breast tissue^17,18^. Besides, the ultrasound artifacts from speckle noise and shadows can also interfere with segmentation. Regarding the above issues, we propose a three-path U-structure network (TPUNet) for breast tumor segmentation. Specifically, we design a three-path encoder to extract multiscale features, and an attention-based feature fusion block (AFF Block) to fuse features extracted from different encoding paths. Moreover, we introduce a hybrid loss for reducing false negative areas and refining the segmentation boundary.

Our contributions are summarized as follows:

- We design a three-path U-structure network (TPUNet) for breast ultrasound segmentation. Different from traditional convolutional frameworks with only a single encoder, we design three independent encoding paths. Each path has a different number of convolutional and pooling layers, corresponding to the extraction of features at different scales, respectively. The model can efficiently deal with objects of various scales thanks to this structure.
- We design an attention-based feature fusion block (AFF Block) to fuse feature maps extracted from different paths. The AFF Block contains two sub-modules: the spatial attention sub-module helps to focus on small targets that are easily overlooked, while the channel attention sub-module learns the importance between different channels. The AFF Block encourages competition between different paths to merge into more salient features.
- We further introduce deep supervision to guide different paths to capture the effective features under the corresponding receptive field, and a hybrid loss for reducing false negative regions and refining boundary segmentation.

The remaining parts of the paper are divided into the following sections: Section “Related works” assesses the related work of medical image segmentation. Section “Methods” interprets specific details of our Three-Path U-structure Network (TPUNet). Section “Experimental setup” describes the experimental details and settings. The experiment’s results are presented in section “Results and discussion”, along with a discussion of important findings and observations. Section “Conclusion” summarizes the contributions and findings of this study.

## Related works

### Encoder–decoder structure

Most segmentation models are inspired by the encoder–decoder structure. Ronneberger et al.^14^ retained abundant feature channels during upsampling, making it a symmetric encoder–decoder structure, which makes UNet transfer advanced semantic features to higher-resolution layers. Zhou et al.^19^ redesigned the skip pathways to have a nested and dense structure. Feature maps in the encoder are upsampled at different stages and transmit contextual information to each other through dense connections in an effort to gradually close the enormous semantic gap between deep and shallow feature maps through a variety of decoding paths. Huang et al.^20^ proposed a modified U-structure to capture abundant contextual information from full scales. Unlike UNet, which directly concatenates feature maps on the identical scale, UNet3+ integrates feature maps in the decoder with feature maps of various scales in the encoder. This structure allows UNet3+ to capture abundant contextual information from full scales. Gu et al.^21^ constructed a dense module to expand the corresponding receptive field sizes by using dilated convolution, and a multiscale pooling operation to integrate contextual information. Jia et al.^22^ proposed a densely connected multiscale residual module to extract and fuse information, and a pixel-based attention module to produce a weighted map for the extracted feature map. Ibtehaz et al.^23^ replaced the larger convolution with a succession of $3\times 3$ convolutions, which can achieve a larger receptive field without requiring additional computation. However, while each of these approaches proposes its own scheme to deal with the multiscale problem, the majority of them are unable to deliver a satisfactory outcome. We suggest a three-path encoder in this study to capture multiscale contextual information. The structure with multiple encoding paths allows our model to easily cope with objects at different scales.

### Attention mechanism

Channel attention, spatial attention, and self-attention are the three primary types of attention mechanisms. Channel attention weights different channels according to their respective importance^24,25^. Spatial attention guides the network to concentrate on the objects that are small and easily overlooked, such as boundaries and small targets^26–28^. Self-attention focuses on information from a long distance, which is difficult for CNNs^29–33^. Oktay et al.^28^ introduced an attention-based gating system that can focus on targets of varying shapes and sizes. Woo et al.^26^ proposed an attention module that progressively infers attention mappings over the channel and spatial dimensions. Chen et al.^27^ introduced a hybrid adaptive attention module that generates corresponding attention maps under different receptive fields. Vaswani et al.^29^ first proposed Transformer, which obtains self-attention between different sequences by encoding operations. Dosovitskiy et al.^30^ designed the Vision Transformer, which cuts the image into small chunks and encodes them to be fed into the Transformer. Based on this, Chen et al.^31^ designed TransUnet to put the underlying features extracted by CNNs into Transformer to enhance the relationship from a long distance. Liu et al.^32^ proposed the swin-Transformer, which calculates self-attention within the windows and shifts the window in order to interact with the information in different windows. Cao et al.^33^ designed a UNet-like symmetric structure by replacing convolutional operations with the swin-Transformer, which achieves excellent performance with less computational expense. In this study, we build an attention-based feature fusion block (AFF Block) to weight and combine the features extracted from different paths. This allows different encoding paths to form competing relationships, which helps to extract more salient features.

## Methods

**Figure 2:**
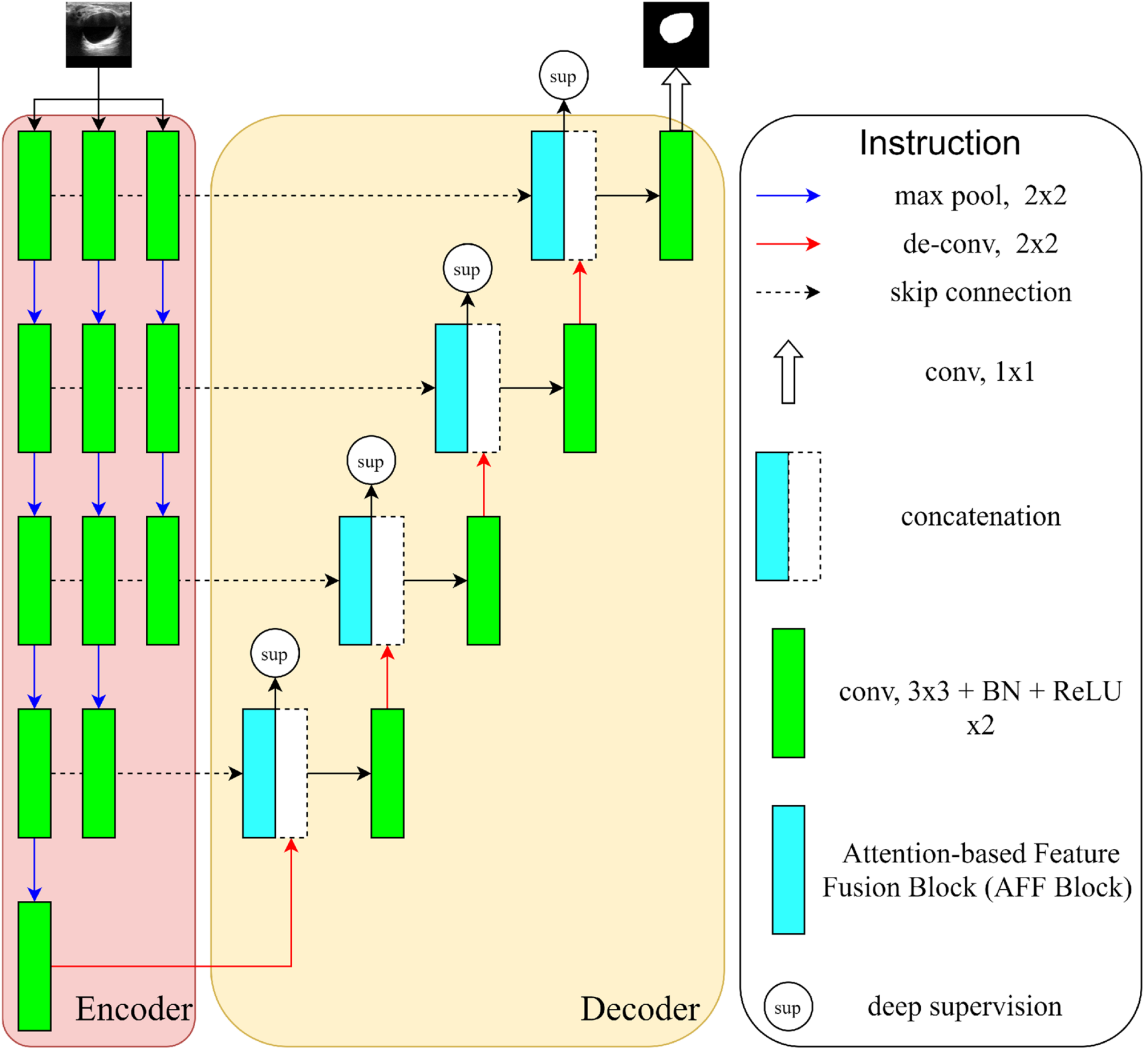
The description of the three-path U-structure network (TPUNet). Three-path encoder extracts multiscale features from different paths and fuses them through the attention-based feature fusion block (AFF Block). The deep supervision is further introduced to guide the model.

Breast cancer prevention and therapy greatly benefit from breast ultrasound segmentation. In this section, we show the main architecture and details of our TPUNet. As shown in Fig. 2, the suggested version consists of a three-path encoder, the attention-based feature fusion block (AFF Block), and deep supervision. The method extracts multiscale features by using multiple independent encoding paths with different depths, and achieves multiscale feature fusion through the AFF blocks. Deep supervision and a hybrid loss are used to further guide and refine the segmentation.

### Three-path encoder

The lesion areas of breast ultrasound images often vary greatly in size, shape, and location, requiring the capacity of the model to extract multiscale features. Traditional CNNs extract features by stacking numerous convolutional and pooling layers. However, repeated stacking of convolutional and pooling layers will destroy spatial information. As illustrated in extensive literature^34–36^, shallow feature maps contain low-level semantic features but detailed texture information, while deep feature maps contain high-level semantic features but abstract signal information. The deeper structure is helpful for segmentation tasks, but spatial information is gradually diluted in the top-down encoder. Therefore, the decoder needs to acquire enough information from the encoder to reconstruct the high-resolution segmentation maps. This single encoder–decoder structure makes it difficult for these models to explore advanced semantic and spatial information at different scales simultaneously, which is not conducive to multiscale feature fusion.

Different from those methods, we design a three-path encoder with three independent encoding paths to deal with multiscale problems in BUS images. Specifically, the images are fed into three independent encoding paths and share the same decoder. As depicted in Fig. 2, the inner path has the fewest convolutional layers, which enables it to effectively preserve texture and spatial information. We contend that small targets are easily lost in continuous convolutional and pooling layers, and the inner path can help preserve the information of small targets. In our method, from inner to outer paths, the number of convolution and pooling layers rises gradually. The inner path stores detailed texture features, while the outer path extracts feature information with advanced semantics. The decoding path starts with the outermost encoding path. The features extracted from all encoder paths are fused through the AFF block and gradually supplied to the decoder.

### Attention-based features fusion block

**Figure 3:**
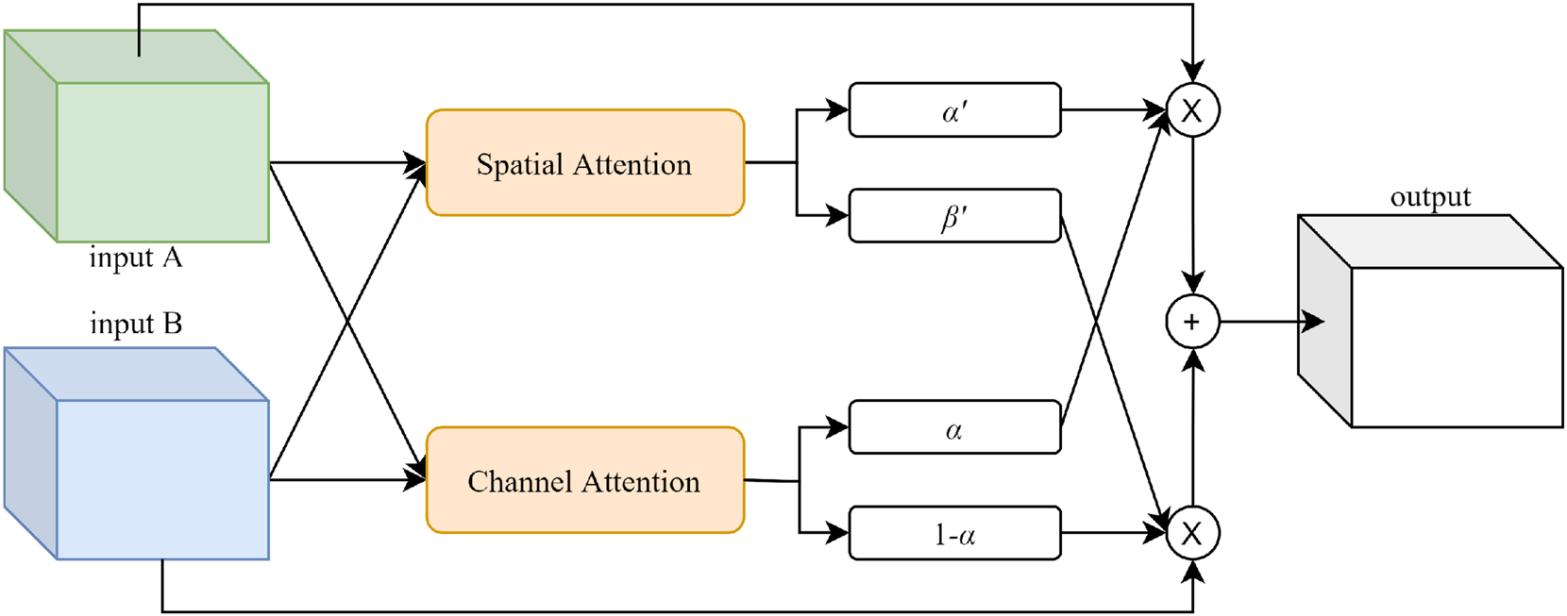
The description of the attention-based feature fusion block (AFF Block) for two inputs.

As illustrated in Fig. 3, the feature maps extracted from different paths are fed into the attention-based features fusion block (AFF Block) to produce more salient feature maps by weighted fusion. The AFF Block is consisted of two sub-modules: the channel attention sub-module and the spatial attention sub-module.

**Figure 4:**
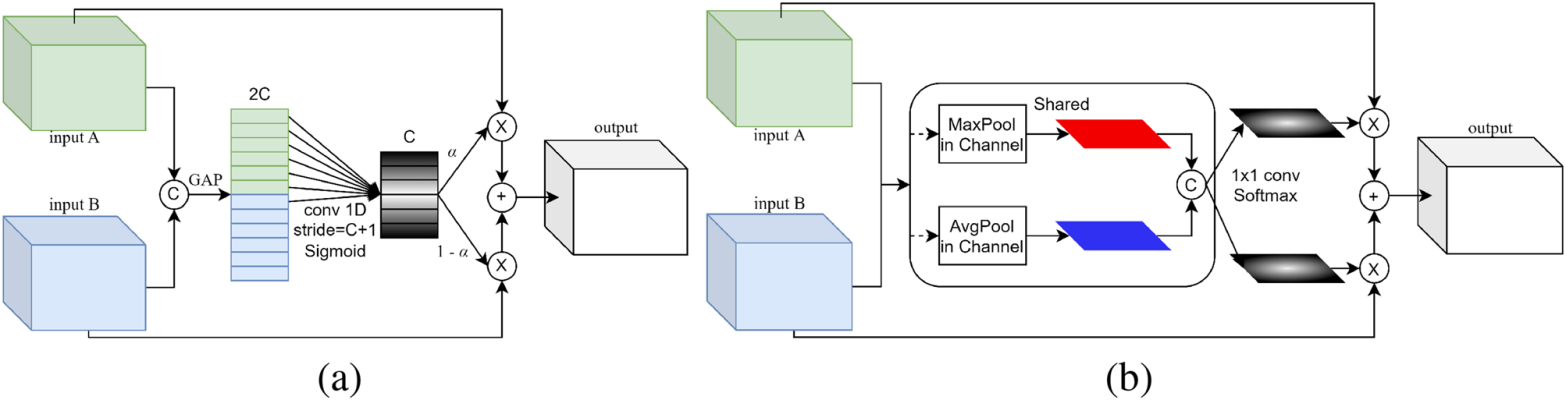
The description of (**a**) the channel attention sub-module and (**b**) the spatial attention sub-module.

In the channel attention sub-module, as shown in Fig. 4a, we perform global average pooling (GAP) for two inputs A and B, and concatenate them together. Then we use 1D convolution with the stride of C+1 and a Sigmoid activation function to produce the attention weights:1After the Sigmoid activation function, $\alpha$ is a vector with the value from 0 to 1, which has the same number of channels as the inputs. We let $\alpha$ multiply the input A to represent the channel-weighted maps of A. Similarly, $1-\alpha$ represents the importance of input B between each channel, which increases the competition between A and B. We use the combination of A and B as the output, and the output is described as follows:

2 $$\begin{aligned} output = \alpha *A+(1-\alpha )*B, \end{aligned}$$

The channel attention sub-module can help us to weight the feature maps extracted from different paths according to their importance and fuse them into more salient feature maps.

In the spatial attention sub-module, as shown in Fig. 4b, we also start with average pooling (AP) and maximum pooling (MP), but the difference is that this time the pooling operation is done on the channel axis. We concatenate two outputs and use $1 \times 1$ convolution to change channels to 1. We concatenate the results from inputs A and B, and use a Softmax activation function to produce the attention weights:3where Conv1 and Conv2 represent two independent convolutions. We let $\alpha$ and $\beta$ represent the spatially weighted maps of A and B, respectively. As a result of the Softmax activation function, the sum of the values of each pixel point between $\alpha$ and $\beta$ is 1, which represents the importance of each pixel point information between A and B. Similarly, this makes A and B compete with each other. We let the combination of A and B be the output, and the output is described as follows:

4 $$\begin{aligned} output = \alpha *A+\beta *B, \end{aligned}$$

The spatial attention sub-module can help us concentrate on objects that are easy to overlook.

AFF Block integrates the advantages of the above sub-modules, which weight the feature maps in the spatial and channel dimensions at the same time, and the output is described as follows:

5 $$\begin{aligned} output = \alpha *\alpha '*A+(1-\alpha )*\beta '*B, \end{aligned}$$

**Figure 5:**
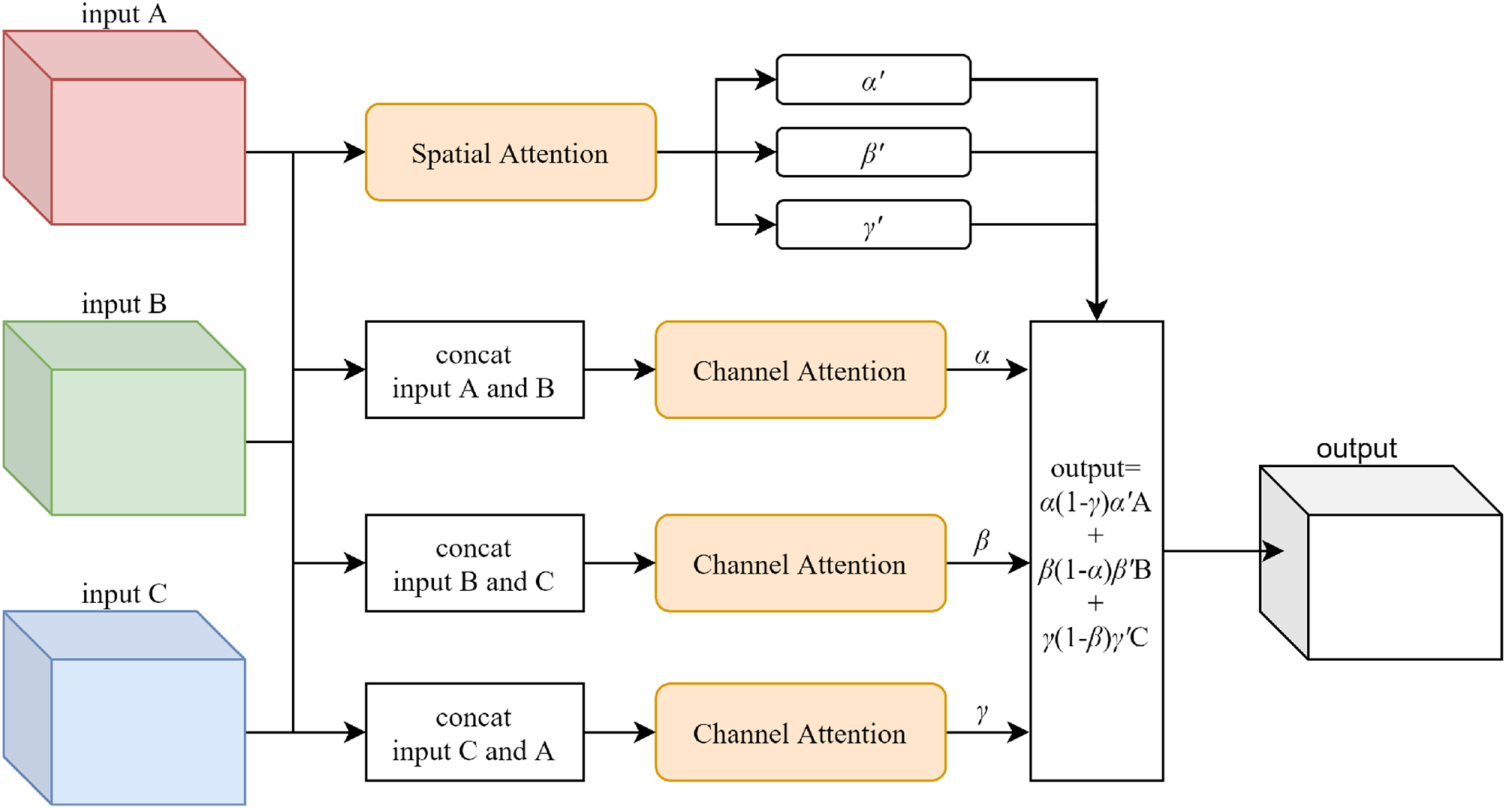
The description of the attention-based feature fusion block (AFF Block) for three inputs.

$\alpha$ is computed from the channel attention sub-module. $\alpha '$ and $\beta '$ are computed from the spatial attention sub-module. Similarly, for stages with three inputs, we calculate the output as depicted in Fig. 5, and the output is described as follows:

6 $$\begin{aligned} output = \alpha *(1-\beta )*\alpha '*A+\beta *(1-\gamma )*\beta '*B+\gamma *(1-\alpha )*\gamma '*C, \end{aligned}$$

where $\alpha , \beta , \gamma$ are computed by concatenating different feature maps and feeding them into the channel attention sub-module, and $\alpha ', \beta '$, $\gamma '$ are computed by the spatial attention sub-module.

### Deep supervision and loss function

Deep supervision is further used in the TPUNet to guide the model to direct effective contextual information from full scales. The deep supervision connects the intermediate layer directly to the loss function, which effectively improves the information flow and gradient flow, and provides powerful guidance. The feature maps are fed into a $3 \times 3$ convolution at each level of the decoder and upsampled to the original image size. To determine the loss for the output of each deep supervision, we employ the cross-entropy loss function, which is described as follows:

7 $$\begin{aligned} L_{deepsup} = \sum _{i=1}^{4} \lambda _i* \left( \frac{1}{N}\sum (-g_t*log(p_i)-(1-g_t)*log(1-p_i))\right) , \end{aligned}$$

$p_i$ is the output of deep supervisions in different stages and upsampled to the original image size. $g_t$ is the ground truth. N is the number of pixels and $\sum$ is the sum for all pixels. $\lambda _i$ is the weight between deep supervisions in different stages. As illustrated in Fig. 2, the deep supervision loss in each stage of the network has the same importance, so we set each $\lambda _i$ to 0.1.

For segmentation tasks, cross-entropy and dice loss are frequently employed as loss functions. Cross-entropy loss describes the difference between the prediction and the ground truth. However, in medical segmentation, the background typically makes up a much larger portion of the target, which leads to an imbalance between the background and targets. Small targets make this more obvious because loss functions will focus on a larger portion of the background while disregarding targets. Dice loss describes the overlap region between the prediction and the ground truth. In general, Dice loss is typically more concerned with the target than the background since the gradient of targets is typically greater than the gradient of backgrounds. Current methods always combine Cross-Entropy loss and Dice loss to balance the attention between the target and background, but those methods usually ignore the false negative (FN) regions. As for most medical image segmentation tasks, FN regions are typically associated with undiagnosed lesions, which frequently results in incorrect diagnoses and illness recurrence.

In this work, we introduce Focal loss^37^ to balance attention between targets and background, as well as simple and hard samples. Focal loss is described as:

8 $$\begin{aligned} L_{focal} =\frac{1}{N}\sum (-\alpha _t(1-p_t)^{\gamma }g_tlog(p_t)-(1-\alpha _t)p_t^{\gamma }(1-g_t)log(1-p_t)), \end{aligned}$$

where N is the number of pixels and $\sum$ is the sum for all pixels. $p_t$ is the output of the network and $g_t$ is the ground truth. $\alpha _t$ is used to balance attention between targets and background. In this paper, $\alpha _t$ is set to 0.8. $\gamma$ is used to balance easy samples and hard samples and we set $\gamma$ to 2.

To further reduce medical accidents, we use Focal Tversky loss^38,39^. Focal Tversky loss is described as:

9 $$\begin{aligned} L_{focal\_tversky}=(1-T(A,B))^{\gamma }, \end{aligned}$$

where *T*(*A*, *B*) is defined as :

10 $$\begin{aligned} T(A,B) = \frac{TP}{TP+\alpha FP+\beta FN}=\frac{\sum p_{0i}g_{0i}}{\sum p_{0i}g_{0i}+\alpha \sum p_{0i}g_{1i}+\beta \sum p_{1i}g_{0i}}, \end{aligned}$$

where $p_{0i}$ and $g_{0i}$ are the output of the network and ground truth. $p_{1i}=1-p_{0i}$ and $g_{1i}=1-g_{0i}$. $\sum$ is the sum for all pixels. $\alpha$ and $\beta$ are used to balance FP and FN, we set $\alpha$ to 0.3 and $\beta$ to 0.7 in this paper. $\gamma$ is used to balance easy samples and hard samples, and we set $\gamma$ to 2 in this paper.

We find that current methods rarely have a loss function that focuses exclusively on the boundary. Therefore, we introduce Hausdorff Distance loss^40–42^ to enhance the boundary of tumors. Hausdorff Distance loss is described as:

11 $$\begin{aligned} L_{HD} = \frac{1}{N}\sum (g-p)^2*(d(p)^2+d(g)^2), \end{aligned}$$

where *p* is the output of the network and *g* is the ground truth. N is the number of pixels and $\sum$ is the sum for all pixels. $d(*)$ is the function to compute the distance map.

In conclusion, we develop a segmentation loss to refine breast ultrasound segmentation. The segmentation loss is defined as:

12 $$\begin{aligned} L_{seg} = (L_{focal}+L_{focal\_tversky})+\alpha *L_{HD}, \end{aligned}$$

where $\alpha$ is used to balance attention between regions and boundaries. Because the Hausdorff Distance loss is unstable in the early epochs of training, the initial value of $\alpha$ is set to 0 and increases by 0.005 after each epoch but not more than 1, which turns attention from regions to boundaries gradually.

In summary, the hybrid loss function we designed is as follows:

13 $$\begin{aligned} L_{total} = L_{deepsup}+\lambda *L_{seg}, \end{aligned}$$

where $\lambda$ is used to balance deep supervision and segmentation loss. We set $\lambda$ to 0.6 to ensure that the network pays more attention to the segmentation loss. The deep supervision loss is used to guide the training of the internal modules and to improve gradient flow. The segmentation loss makes the network learn the regional features of breast tumors in the early epochs and refine the boundaries gradually in the later epochs.

## Experimental setup

### Dataset and pre-processing

We put our method to the test on the Breast Ultrasound Images Dataset (Dataset BUSI^43^). The data consisted of breast ultrasound scans performed on people from 25 to 75 years old, which were gathered from 600 female patients in 2018. Due to the protection of patient privacy, this dataset does not provide specific age distributions, but this does not affect the main points and conclusions of our study. All images were pre-processed by radiologists and screened for classification as normal (n = 133), benign (n = 437), and malignant (n = 210) datasets, provided by Baheya Hospital, Cairo, Egypt.

We exclude 16 samples from the benign tumor dataset that require multiple segmentation objectives. The remaining 421 pictures are randomly divided into a train set (n = 253), a validation set (n = 84), and a test set (n = 84). In the malignant tumor dataset, we also remove 1 sample that requires multiple segmentation objectives and use all remaining 209 images, dividing them into a train set (n = 125), a validation set (n = 42), and a test set (n = 42) randomly.

We can’t directly feed them into the model because the size between different pictures is inconsistent. We take the longer side of the picture as the edge length and fill the picture with black to make it square. Then we resize the pictures to $224 \times 224$. We apply data augmentation, including random flip, random rotation, and normalization, to further improve the generalization performance and robustness of our model.

### Implementation details

We train our framework on the RTX A6000 48G and the Ubuntu operating system. We train models on benign and malignant tumor datasets. We monitor the mean Intersection over Union (mIoU) and the mean 95% Hausdorff Distance (HD95) on respective validation sets, saving model parameters when mIoU is promoted. Additionally, we employ the Adam optimizer with a 0.003 starting learning rate, halving the learning rate when there is no promotion within 30 epochs on the validation set. All results are scored on the respective test sets.

### Evaluation metrics

We evaluate the segmentation performance of the methods by calculating the Intersection over Union (IoU) and 95% Hausdorff Distance (HD95), which are defined as follows:

14 $$\begin{aligned} IoU&=\frac{A\cap B}{A\cup B}, \end{aligned}$$

15 $$\begin{aligned} HD95&=95\% max(h(\partial A,\partial B),h(\partial B,\partial A)), \end{aligned}$$

where *A* and *B* are two regions and $\partial A$ and $\partial B$ are their boundary curves. $h(\partial A,\partial B)$ and $h(\partial B,\partial A)$ are the distance functions between the two curves, which is defined as:

16 $$\begin{aligned} h(\partial A,\partial B)=\mathop {max}\limits _{a\in \partial A}{\mathop {min}\limits _{b\in \partial B}\Vert a-b \Vert },\ h(\partial B,\partial A)=\mathop {max}\limits _{b\in \partial B}{\mathop {min}\limits _{a\in \partial A}\Vert b-a \Vert }, \end{aligned}$$

where *a* and *b* are points in the boundary curves $\partial A$ and $\partial B$, respectively.

## Results and discussion

### Ablation study

To confirm the validity of each module in our proposed method, we conduct an ablation experiment. We run the experiment with the same setting for a fair comparison. The first line is our primitive TPUNet, with no added any modules and no deep supervision. In the second line, we add deep supervision to the model. In the third line, we add the deep supervision and channel attention sub-modules to the model. In the fourth line, we add the deep supervision and spatial attention sub-modules to the model. In the fifth line, we add the deep supervision and AFF modules to the model. In the last line, we use the hybrid loss function Eq. (13) to train. The results are illustrated in Table 1, while all results are selected with the largest common domain by post-processing. As illustrated in Table 1, the original model has multiple encoding paths, which makes it difficult to optimize. However, the accuracy was dramatically improved after adding deep supervision, which proves the superiority of our model. Besides, the performance of the model can be further improved with the addition of the channel and spatial attention sub-modules. Our AFF Block combines the advantages of the channel and spatial attention sub-modules, and achieves better results than both. On top of this, to improve the segmentation even further, our final suggested model also includes a hybrid loss function. The results of the experiments show that our final strategy produces the best outcomes.

**Table 1:** Results of ablation study. The best results are highlighted in bold.

	Benign		Malignant	
	mIoU $\uparrow$	mHD95 $\downarrow$	mIoU $\uparrow$	mHD95 $\downarrow$
TPUNet (raw)	0.69	17.42	0.5431	35.38
TPUNet (+deepsup)	0.7283	11.62	0.5679	31.39
TPUNet (+deepsup+chatt)	0.7435	15.33	0.5747	32.88
TPUNet (+deepsup+spatt)	0.7366	12.71	0.5764	31.26
TPUNet (+deepsup+AFF)	0.7451	15.24	0.5858	28.68
TPUNet (proposed)	**0.7531**	**11.55**	**0.6079**	**27.77**

### Comparisons with the state of the art

**Figure 6:**
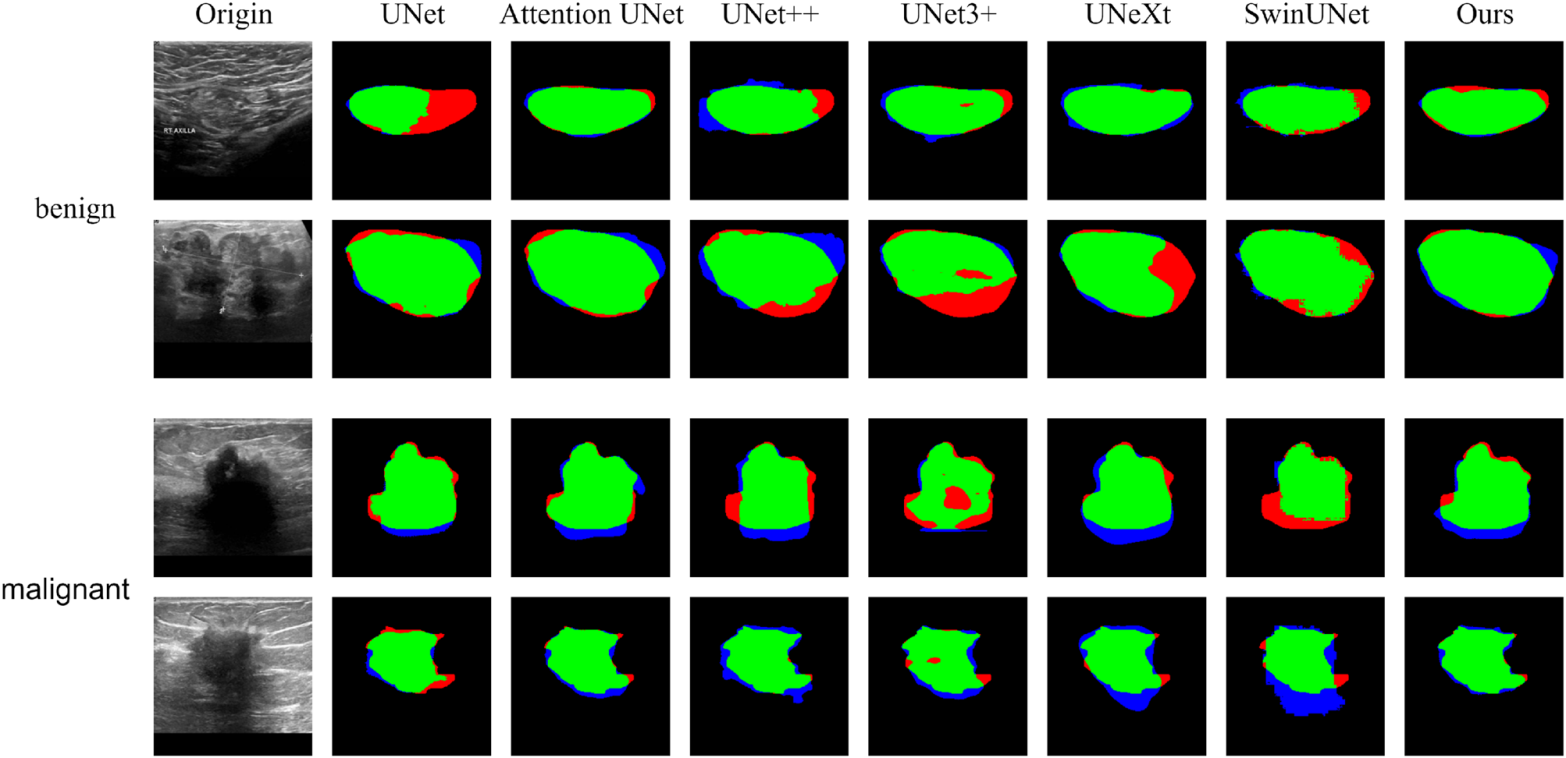
Comparison between different methods for benign tumor and malignant tumor segmentation on the BUSI dataset. Green areas: the true positive (TP); Blue areas: the false positive (FP); Red areas: the false negative (FN). The combination of the green and red areas is the ground truth. The combination of the green and blue areas is the prediction.

We compare our TPUNet with several state-of-the-art methods: UNet^14^, Attention U-Net^28^, UNet++^19^, UNet3+^20^, UNeXt^44^ and Swin-UNet^33^. All methods are optimized by the training environment suggested in their own paper. The results are illustrated in Table 2, and the segmentation maps are depicted in Fig. 6. As illustrated in Table 2, our TPUNet outperforms other approaches on both mIoU and HD95, which suggests that it can clearly outline the boundaries of the tumor, which is challenging for other methods to do. Moreover, as can be observed in Fig. 6, for benign tumors of varying sizes, shapes, and locations, our model handles them brilliantly and stably, while for malignant tumors with blurred boundaries that are difficult to segment, our method can segment the smoothest and most coherent boundaries. Besides, our method can produce the smallest FP and FN regions, which can effectively reduce medical accidents.

**Table 2:** Results of comparisons with the state of the art. The best results are highlighted in bold.

		UNet	Attention UNet	UNet++	UNet3+	UNeXt	SwinUNet	Ours
Benign	mIoU $\uparrow$	0.7185	0.7234	0.7308	0.7398	0.6193	0.7378	**0.7531**
	mHD95 $\downarrow$	16.44	11.99	17.75	14.43	17.62	12.16	**11.55**
Malignant	mIoU $\uparrow$	0.5297	0.5607	0.5822	0.5804	0.5465	0.5969	**0.6079**
	mHD95 $\downarrow$	35.34	31.09	29.69	32.48	32.28	27.91	**27.77**

Numerous segmentation methods have been developed recently to address multiscale issues. However, most of them only attach some specially designed modules or skip connections. Despite achieving excellent results, those approaches are still limited by the single encoder. In this work, we design a three-path U-structure network (TPUNet) to solve multiscale problems. The three independent encoding paths with different depths correspond to the extraction of features at different scales. In addition, we design the attention-based feature fusion block (AFF Block) to further facilitate the competition and fusion of features. The AFF Block integrates the advantages of the spatial and channel attention sub-modules. The AFF Block encourages different paths to compete with each other and makes a weighted fusion of feature maps from different paths in the channel and spatial dimension to obtain more salient features. Thanks to this structure, our method can extract abundant contextual information on different scales.

Most existing models ignore the importance of boundaries and FN regions, which increases the risk of medical accidents. Subtle differences in boundaries can make an extreme impact on radiologists’ policy decisions, while FN regions imply undetected nidus, which usually leads to misdiagnosis and disease recurrence. We propose a hybrid loss function to further refine segmentation to meet those problems. As shown in Fig. 6, the segmentation maps generated using our method have the smoothest boundary and the smallest FN regions compared to using other competing methods. Besides, we discover that our technique performs better in small organ segmentation across a vast number of studies. We argue it benefits from the structure of multiple coding paths. In the classic encoder–decoder structures, small targets are easy to lose in continuous convolutional and pooling layers. Different from those methods, the three-path encoder architecture proposed in our TPUNet allows us to retain sufficient detailed information in the inner path and to recall and fuse it in the decoder.

## Conclusion

In this paper, we propose a three-path U-structure Network named TPUNet to improve multiscale problems in BUS segmentation. The structure of multiple encoding paths shows a new way to deal with multiscale problems in segmentation tasks. Our approach also provides outstanding performance in the segmentation of tiny targets thanks to the structure of multiple coding paths. The AFF Block we designed can filter and weight multiscale feature maps extracted from different encoding paths, and fuse them into a more representative feature map. Moreover, we further propose a hybrid loss function by introducing Focal loss, Focal Tversky loss, and Hausdorff Distance loss, which can reduce the false negative areas and refine segmentation boundaries gradually. Experimental results show that our suggested strategy produces greater accuracy and smoother borders when compared with previous approaches.

## Data Availability

This study did not include any human clinical images or patient data from restricted datasets. The experiments in this study have been performed in a publicly available dataset (Dataset of Breast Ultrasound Images^43^ *figshare*10.1016/j.dib.2019.104863). All data generated and analysed during this study are available from the corresponding author.
